# Mediating role of systemic immune-inflammation index between heavy metal exposure and hepatic steatosis/hepatic fibrosis: evidence from NHANES 2005–2020

**DOI:** 10.3389/fnut.2025.1566345

**Published:** 2025-05-21

**Authors:** Ningning Wang, Xuying Li, Rui He, Xiujuan Zheng, Mingqi Li, Shijing Nian, Kewei Wang

**Affiliations:** ^1^Center for Endemic Disease Control, Chinese Center for Disease Control and Prevention, Harbin Medical University, Harbin, China; ^2^Department of Preventive Medicine, Qiqihar Medical University, Qiqihar, China; ^3^Institute of Cell Biotechnology, China and Russia Medical Research Center, Harbin Medical University, Harbin, China; ^4^Harbin Municipal Center for Disease Control and Prevention, Harbin, China

**Keywords:** hepatic steatosis, hepatic fibrosis, systemic immune verification index, heavy metal, mediating effect

## Abstract

**Background:**

Moderate heavy metals can lead to the occurrence of liver injury, but the specific mechanism remains unclear.

**Methods:**

This study, based on data from the National Health and Nutrition Examination Survey (NHANES), analyzed associations between 10 heavy metals and hepatic injury in 5,613 adults, with a focus on the mediating role of the Systemic Immune-Inflammation Index (SII). Partial correlation analysis, weighted linear regression, weighted quantile sum (WQS) regression, and mediation effect models were used in the study.

**Results:**

SII showed significant negative correlations with hepatic fibrosis markers (FIB-4: *r* = −0.290; NFS: *r* = −0.382, both *P* < 0.001) but no association with hepatic steatosis indices. Arsenic (As), cobalt (Co), and cesium (Cs) were identified as critical metals linking fibrosis indicators and SII. As mediated its pro-fibrotic effects by completely suppressing SII (OR = 0.0220–0.0581), while Co promoted NFS risk through complete mediation by SII (Q2 vs. Q1 OR = 1.26). Conversely, Cs exhibited anti-fibrotic protectionvia complete positive mediation through SII.

**Conclusion:**

The findings demonstrate that Heavy metals differentially regulate immune-inflammatory pathways to influence hepatic fibrosis progression, providing new evidence for the mechanisms of environmental exposure-induced hepatic injury.

## Introduction

Heavy metal pollution is a major problem faced by every city. The heavy metal pollution index in Nigeria's drinking water was as high as 13,672.74, which was 136.72 times higher than the World Health Organization standard for drinking water (1). Heavy metal pollution is also a problem in Ecuador (2), China (3), Canada (4) and the United States (2–4), and has even been detected in the sparsely populated Antarctic (5). It may be produced by large-scale human activities such as the use of pesticides, internal combustion engines and automobiles, rapid industrialization, and imperfect environmental planning, and so on (6, 7). Heavy metal pollution has begun to attract the attention of all countries, but the management is still insufficient. After heavy metals enter the human body, the hepatic is the first to be affected, resulting in abnormal hepatic function and fibrosis (8–10). With the excessive accumulation of heavy metals, the human brain, lungs, and nerves are further damaged (11–15).What is more serious is that they also show carcinogenic effects, such as arsenic, chromium, lead etc. (16). However, the mechanism by which heavy metals cause hepatic damage has not yet been clarified.

The systemic immune-inflammation index (SII), as a new inflammatory biomarker, can reflect the systemic local immune response and systemic inflammation, and has shown its role in the pathogenesis and prognosis of a variety of diseases, such as coronary heart disease (17, 18), gastric cancer (19, 20), lung cancer (21–23) and so on. Some studies have pointed out that SII may be associated with the risk and severity of various hepatic diseases (24, 25). Alterations in inflammatory markers may result from the toxic effects of harmful metals. For instance, arsenic induces sustained immuno-inflammatory responses in the hepatic and kidneys (26), meanwhile, chronic cadmium (Cd) exposure triggers hepatic oxidative stress, endoplasmic reticulum stress, inflammatory reactions, and proliferation in aged female mice (27). Based on these studies, we hypothesize that SII may play a role in the relationship between heavy metals and hepatic injury. However, the specific role of SII in this regard has not been reported.

The development of hepatic injury has a certain process, from the ectopic accumulation of triglycerides in the cytoplasm of hepatic cells (i.e., hepatic steatosis), forming inflammation and hepatocyte damage [i.e., non-alcoholic steatohepatitis], to progressive fibrosis and development into hepatic cirrhosis, end-stage hepatic disease or hepatic cancer (28). Previously, the most common marker for diagnosing hepatic injury was alanine aminotransferase (ALT), but a single factor had the drawback of poor accuracy. Therefore, in this study, the Fatty Liver Index (FLI) (29), NAFLD hepatic fat score (LFS) (30), and Framingham Steatosis Index (FSI) were selected to reflect the level of hepatic steatosis. The degree of hepatic fibrosis was represented by the nonalcoholic fatty liver disease fibrosis score (NFS), an indicator of hepatic fibrosis (FIB-4). Therefore, we investigated the relationship between metal exposure and hepatic damage-related indicators (FLI, LFS, FSI, FIB-4 and NFS) using data from the National Health and Nutrition Survey (NHANES) of people aged 18–80. And we further studied the mediating role of SII in the relationship between heavy metals and Non-alcoholic Fatty Liver Disease (NAFLD).

## Methods

NHANES is a program of studies designed to assess the health and nutritional status of adults and children in the United States, by using a hierarchical, multi-stage and probabilistic clustering design. Information will be used to assess the health promotion and disease prevention. The study protocol was approved by the National Center for Health Statistics Ethics Review Board, and all participants provided their informed consent.

### Study design and participants

A total of 76,496 participants from five NHANES cycles in 2005–2006, 2007–2008, 2009–2010, 2011–2012, 2013–2014, 2015–2016 and 2017–2020 were included in this study. In NHANES, the heavy metal exposure of participants aged 18–80 was evaluated through urine metabolite analysis. We excluded hepatic injury caused by Hepatitis B Virus (HBV) and Hepatitis C Virus (HCV) (*n* = 859), and those who drank excessively or lacked drinking information (women drank ≥3 cups per day; Men who drink ≥4 cups of alcohol per day or 5 cups or more per month (*n* = 18,220), as well as pregnant women (*n* = 742) and cancer patients (*n* = 6,667), because these conditions can affect hepatic function indicators.

Ten heavy metals (Arsenic [As], Barium [Ba], Cobalt [Co], Cesium [Cs], Molybdenum [Mo], Lead [Pb], Antimony [Sb], Thallium [Tl], Tungsten [Tu] and Uranium [Ur] in urine were analyzed as the main indicators.

NHANES usually adopts stratified sampling or subsample analysis strategies and only conducts specific tests on some participants. Therefore, in this study, 12,829 samples with missing urinary arsenic data were deleted. The number of participants lacking the other nine heavy metals was relatively small. In this study, the median was used to complete the missing information. There were 2 participants with deficient urinary creatinine content. SII can reflect the local immune response and systemic inflammatory response of the human body. A total of 191 were removed due to the lack of SII.

**Figure 1:**
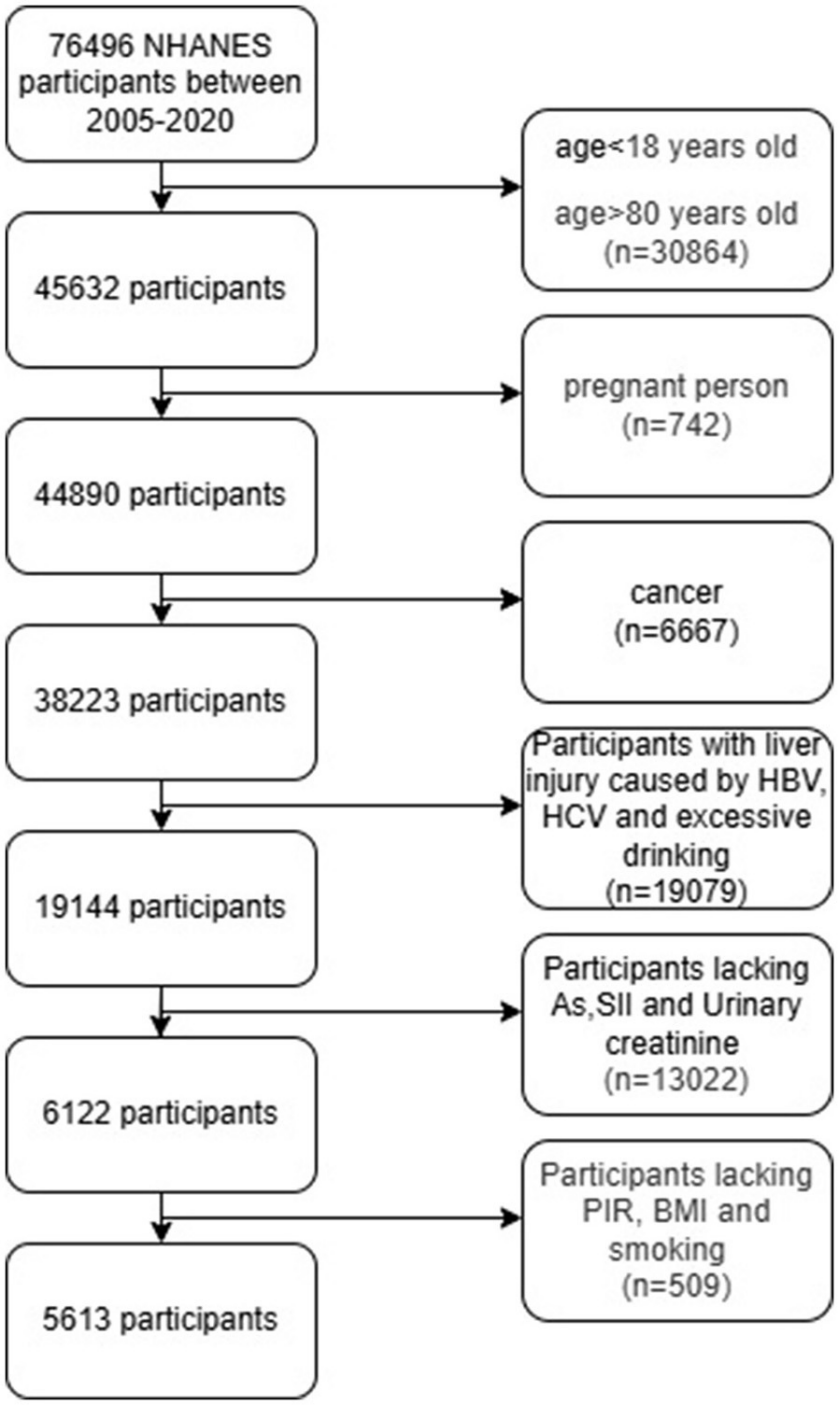
Flowchart of the participants included in the analyses.

Because the influence caused by confounding factors such as age, gender and poverty index (PIR) needs to be adjusted during the calculation process, participants with incomplete basic information were also deleted. Ultimately, there were a total of 5,613 participants with complete indicators of heavy metals, SII and urinary creatinine (Figure 1).

### Evaluation of biomarkers related to hepatic steatosis and hepatic fibrosis

#### Biomarkers of hepatic steatosis

The FLI and LFS demonstrate high diagnostic accuracy as markers of hepatic injury, particularly in metabolically susceptible populations (31, 32). Due to this, FLI and LFS have been widely adopted in scientific research. In 2020, a research team led by Lars Lind compared four diagnostic markers for NAFLD (FLI, Hepatic Steatosis Index [HSI], Lipid Accumulation Product [LAP], and LFS) and found that FLI and LFS were the most suitable for assessing NAFLD. This is because FLI performs better in population-based settings, whereas LFS is optimal in high-risk cohorts (33).

The FSI is another tool for determining NAFLD risk, incorporating age, body mass index (BMI), triglycerides (TG), aspartate aminotransferase (AST), alanine aminotransferase (ALT), diabetes status, and hypertension. A 2024 study reported that when used for NAFLD diagnosis, FSI exhibited discrimination and predictive performance with area under the curve (AUC) values of 0.8421 (95% CI: 0.8314–0.8527) and 0.7093 (95% CI: 0.6863–0.7322),


$$\begin{array}{l} FLI=\frac{e^{\begin{array}{c} 0.953\times ln(TG)+0.139\times BMI\\ +0.781\times ln(GGT)+0.053\times WC-15.745 \end{array}}}{1+e^{\begin{array}{c} 0.953\times ln(TG)+0.139\times BMI\\ +0.781\times ln(GGT)+0.053\times WC-15.745 \end{array}}}\times 100\\ \;LFS=-2.89+1.18\times MS(yes=1,no=0)\\ \;+0.45\times T2DM(yes=2,no=0)\\ \;+0.15\times insulin(mU/L)\\ \;+0.04\times AST(U/L)-0.94\times AST/ALT \end{array}$$

Among them, the diagnostic criteria for metabolic syndrome (MS) are that any three or more of the following adjustments are satisfied:

1. Fasting Blood Glucose (FBG) >100 mg/dL (5.6 mmol/L) or drug treatment for diabetes mellitus.
2. High-density lipoprotein cholesterol (HDL-C) <50 mg/dL in females <40 mg/dL in males or drug treatment for reduced HDL-C.
3. Plasma Triglyceride (TG) >150 mg/dL or drug treatment for raised TG.
4. Waist circumference >88 cm in women or >102 cm in men.
5. Blood pressure >130/85 mmHg or drug treatment for raised blood pressure

$$\begin{array}{l} FLI=-7.981+0.011\times age(years)\\ -0.146\times gender(women=1,men=0)\\ +0.173\times BMI(kg/m^{2})+0.007\\ \times TG(mg/dL)+0.593\times HTN(yes=1,no=0)\\ +0.789\times Hyperglycemia(yes=1,no=0)\\ +1.1\times [ALT/AST\geq 1.33(yes=1,no=0)] \end{array}$$

1. The diagnostic criteria for hypertension are: Systolic Blood Pressure (SBP)≥140 and/or Diastolic Blood Pressure (DBP)≥90, or having been diagnosed with hypertension by a doctor;
2. According to the latest prevention and treatment guidelines, the diagnostic criteria for hyperglycemia are that it can be diagnosed if any of the following conditions are met (34):

① FPG ≥7.0 mmol/L (126 mg/dL) (fasting means not having eaten for at least 8 h).

② The 2-h blood glucose level in the oral glucose tolerance test (OGTT) was ≥11.1 mmol/L (200 mg/dL).

③ Glycated hemoglobin (HbA1c) ≥6.5% (standardized detection methods should be adopted).

④ Random blood glucose ≥11.1 mmol/L (200 mg/dL) and accompanied by typical hyperglycemic symptoms (such as polydipsia, polyuria, and weight loss).

⑤ It had been confirmed as diabetes before the investigation.

#### Biomarkers of hepatic fibrosis

The EASL-EASD-EASO clinical practice guidelines recommend simple non-invasive scores such as the NFS and FIB-4 as part of the diagnostic protocol to exclude advanced fibrosis (35). A re-analysis of 13 studies showed that FIB-4 and NFS could stratify the risk of hepatic-related morbidity and mortality in patients, and their performance was comparable to that of hepatic biopsy (36).


$$\begin{array}{l} \;FIB-4=\frac{Age(years)\times AST(U/L)}{Plateletcount(1000cells/\mu L)\times \sqrt{ALT}}\\ NFS=-1.675+0.037\times age(years)+0.094\\ \times BMI(kg/m^{2})+1.13\\ \times impairedfastingglucose(ordiabetes)(yes=1,no=0)\\ +0.99\times AST/ALTratio-0.013\times platelet(\times 10^{9}/L)\\ -0.66\times albumin(g/dL) \end{array}$$

#### Heavy metal exposure

Inductively coupled plasma mass spectrometry (ICP-MS) is a multielement analytical technique (37). The urine samples were collected and ICP-MS was used to detect 13 elements including As, Ba, Co, Cs, Mo, Pb, Sb, Tl, Tu, Ur, beryllium (Be), cadmium (Cd), and platinum (Pt) in the urine samples. Because Be, Cd, and Pt were missing more, the other 10 heavy metals were analyzed in this study. Urinary creatinine was used to standardize the content of heavy metals to control the bias caused by measurement error. Except for As, the missing values of the other 9 heavy metals were filled with the median, and logarithmic transformation was performed on all heavy metals for subsequent analysis.

#### SII

SII is a good and stable new inflammatory marker that can reflect the local immune response and systemic inflammatory response of the human body. SII was defined as platelet count (10^9^/L) × neutrophil count (10^9^/L)/lymphocyte count (10^9^/L) (38). This comprehensive parameter, which combines peripheral platelet, neutrophils and lymphocytes, more comprehensively reflects the inflammatory state of the body compared with a single inflammatory index (39). Studies have now that SII was associated with a variety of diseases, such as testicular cancer (40), prostate cancer (41), coronary artery disease (42), COVID-19 (43). Since the data distribution of SII did not conform to the properties of a normal distribution, so ln (SII) was used for analysis in this study.

#### Covariates

Demographic variables, such as age (in years), gender, race, education years, marital status, poverty to income ratio, smoking, and drinking, were collected through interviewers assisted interviews. Gender, race/ethnicity, gender and smoking status were grouped by the respondents. Age was calculated in years and was divided into three groups (18–39, 40–59 and ≥60 years old) for subsequent analysis. This study divided the years of education into three groups: ≤ 12 years, 13–14 years, and ≥15 years. Socioeconomic status is a disease risk factor that is easily overlooked. Low household income is an indicator to measure socioeconomic status and may indicate a greater risk of diseases. The research participants were classified into low poverty ( ≤ 1), poverty (1–3), and high poverty (>3) based on PIR. The harm of smoking is obvious to all, but there are still people who smoke. In this study, the subjects were divided into smokers, quitters and never smokers for corrective analysis.

#### Statistical analysis

A total of 76,496 people from the NHANES dataset were collected. According to strict inclusion and exclusion criteria, a total of 5,613 people participated in the final calculation. Considering the complex, multi-stage and multi-level sampling methods used in the NHANES database, we included weighted variables in accordance with the instructions for use provided by the data providers. The weight for the period from 2005 to 2016 was WTSA2YR/6, and the weight for the period from 2017 to 2020 (~1.6 two-year periods) was WTSA2YR/1.6. Weighted non-parametric tests, *t*-tests, and analysis of variance were used to compare the differences in biomarkers of hepatic steatosis and hepatic fibrosis at different baseline levels. Weighted regression was used to analyze the correlation analysis of hepatic fibrosis indicators after overall and quartile grouping of heavy metals. Since multiple inter-group comparisons were required in the weighted regression of quartile grouping, the *P-*value was corrected by Bonferroni.

The effect of combined exposure to heavy metals on hepatic fibrosis was analyzed by WQS (44). The WQS regression model is usually expressed as: g(μ) = β0+β1(∑ω*iqi*)+*z*′ϕ. Among them: μ is the expected response; β0 is the intercept; *q*_*i*_ is the quantile of the *i*-th exposed variable; *w*_*i*_ is the weight of the *i*-th exposed variable (0 ≤ *w*_*i*_ ≤ 1 and ∑*w*_*i*_ = 1); β1 is the coefficient of the comprehensive index; *z* is the covariate vector; ϕ is the covariate coefficient vector. In this study, the WQS model was implemented in R using the “gWQS” package. We assumed that the effect of each exposure in the mixture in the study is in the positive direction. The parameters were set to gwqs(FIB4~ wqs, mix_name = name, data = data, *q* = 4, validation = 0.6, *b* = 100, b1_pos = TRUE, b_constr = FALSE, seed =1,003).

Restricted Cubic Splines (RCS) fits data through piecewise polynomials to automatically capture non-linear trends. There is no need to pre-assume the function form (such as linear or quadratic), and the relationship curves between variables and outcomes can be directly plotted (45). Therefore, in this study, the “rms” package of R software was further utilized. The effects of As, Co, and Cs on FIB-4, NFS, and SII were analyzed by weighted RCS.

Mediation analysis is often used to study how an independent variable (*X*) affects the dependent variable (*Y*) through a mediation variable (*M*). For this reason, mediation analysis was also used in this study, with the aim of exploring the mediating effect of SII in the correlation process among As, Co, Cs, and FIB-4, NFS, and it was achieved through the “mediation” package.

All data analyses were conducted using R version 4.4.3 (R Foundation for Statistical Computing, Vienna, Austria). A two-sided *p-*value was below 0.05. When Bonferroni correction was performed, the two-sided *p-*value was below 0.05/4, that is, the *p-*value was 0.0125.

## Result

### NAFLD correlation index distribution among different participant feature groups

Among 5,613 adults, based on the demographics of the study, Weighted analysis was performed to compare Hepatic steatosis related biomarkers (FLI, LFS, FSI) and Hepatic fibrosis related biomarkers (FIB-4, NFS) in Table 1.

**Table 1:** Characteristics of NHANES 2005–2020 weighted sample and *P*-value.

**Characteristic**	**FLI (median [IQR])**	**LFS (median [IQR])**	**FSI (median [IQR])**	**FIB-4 (median [IQR])**	**NFS [mean(SD)]**
All	53.6 [20.5,84.7]	−1.17 [−2.11,0.39]	−0.99 [−2.37,0.50]	0.86 [0.59,1.27]	−0.57 (1.31)
**Age**					
18–39	37.06 [11.30, 79.09]	−1.69 [−2.35, −0.14]	−2.06 [−3.09, −0.35]	0.56 [0.44, 0.71]	−1.50 (1.10)
40–59	62.38 [26.09, 86.97]	−0.88 [−2.05, 0.65]	−0.64 [−2.00, 0.94]	0.94 [0.75, 1.17]	−0.70 (1.05)
≥60	63.66 [31.75, 87.10]	−0.33 [−1.81, 1.00]	−0.35 [−1.46, 0.91]	1.51 [1.19, 1.88]	0.30 (1.12)
*P*	<0.001	<0.001	<0.001	<0.001	<0.001
**Gender**					
Male	63.09 [30.61, 86.78]	−0.56 [−1.84, 1.07]	−0.76 [−2.11, 0.66]	0.83 [0.58, 1.22]	−0.71 (1.24)
Female	39.58 [13.01, 81.76]	−2.30 [−2.58, −1.91]	−1.21 [−2.66, 0.59]	0.83 [0.56, 1.18]	−0.46 (1.28)
*P*	<0.001	<0.001	0.034	0.105	0.005
**Race**					
White	52.79 [18.08, 84.80]	−1.29 [−2.26, 0.43]	−0.96 [−2.48, 0.59]	0.87 [0.59, 1.27]	−0.52 (1.28)
Black	52.23 [19.23, 87.24]	−1.01 [−2.10, 0.24]	−0.65 [−2.06, 0.78]	0.76 [0.54, 1.12]	−0.39 (1.24)
Others	54.47 [21.22, 81.90]	−0.91 [−1.85, 0.99]	−0.83 [−2.18, 0.79]	0.72 [0.52, 1.02]	−0.96 (1.17)
*P*	0.721	0.023	0.083	<0.001	<0.001
**Education years**					
≤ 12 grade	54.21 [25.01, 85.10]	−0.78 [−2.09, 0.95]	−0.76 [−2.17, 0.63]	0.81 [0.56, 1.20]	−0.80 (1.37)
13–14 grade	65.56 [26.96, 89.16]	−0.40 [−1.84, 1.33]	−0.52 [−1.68, 1.33]	0.81 [0.52, 1.18]	−0.55 (1.26)
≥15 grade	48.97 [16.31, 83.35]	−1.51 [−2.25, 0.29]	−1.23 [−2.54, 0.42]	0.84 [0.58, 1.20]	−0.59 (1.24)
*P*	<0.001	0.002	<0.001	0.223	0.192
Marital	57.92 [22.86, 85.13]	−0.88 [−2.05, 0.68]	−0.71 [−2.07, 0.71]	0.87 [0.64, 1.25]	−0.61 (1.19)
*P*	<0.001	0.01	0.011	<0.001	0.836
**PIR**					
≤ 1	55.10 [17.44, 86.38]	−1.05 [−2.04, 1.00]	−0.84 [−2.55, 0.80]	0.63 [0.47, 1.00]	−0.82 (1.25)
1–3	56.50 [19.30, 86.56]	−0.90 [−2.08, 0.75]	−0.73 [−2.10, 0.89]	0.81 [0.56, 1.21]	−0.52 (1.36)
≥3	50.89 [18.72, 83.13]	−1.31 [−2.18, 0.42]	−1.05 [−2.53, 0.45]	0.87 [0.62, 1.22]	−0.62 (1.20)
*P*	0.188	0.28	0.081	<0.001	0.062
**Smoking**					
Smokers	51.53 [16.13, 83.46]	−1.50 [−2.30, 0.09]	−1.15 [−2.70, 0.48]	0.73 [0.53, 1.00]	−0.93 (1.13)
Former smokers	66.31 [25.86, 89.36]	−0.29 [−1.99, 1.29]	−0.33 [−1.87, 1.16]	0.93 [0.68, 1.37]	−0.40 (1.22)
Never smokes	48.26 [17.25, 80.94]	−1.38 [−2.16, 0.35]	−1.21 [−2.52, 0.38]	0.81 [0.54, 1.18]	−0.64 (1.31)
*P*	<0.001	0.005	<0.001	<0.001	<0.001
**Drinking**					
Never drinks	56.05 [20.03, 84.80]	−0.85 [−2.03, 0.56]	−0.57 [−1.99, 0.91]	0.87 [0.59, 1.27]	−0.50 (1.28)
Light drinkers	49.63 [16.85, 83.86]	−1.57 [−2.34, 0.42]	−1.46 [−2.79, 0.21]	0.74 [0.54, 1.05]	−0.85 (1.19)
*P*	0.196	0.007	<0.001	<0.001	0.001

Weighted baselines were used to compare the differences in hepatic injury-related indicators among different baseline level groups. The comparison of FLI, LFS, FSI, and FIB-4 were conducted using non-parametric tests, and NFS was performed using fangchafenx (age, race, education years, PIR, and smoke) or t-tests (gender, marry, and drink).

Of all the participants, the older the subjects had higher level of NAFLD related index including Hepatic steatosis-related biomarkers (FLI, LFS, FSI), and Hepatic fibrosis-related biomarkers (FIB-4, NFS), *P* < 0.001. The median level of Hepatic steatosis related biomarkers in males was significantly higher than that in females, while that in NFS was significantly lower than that in females (*P* < 0.01). LFS of Black people was higher than that of White people (*P* = 0.023), however, the conclusions of biomarkers for hepatic fibrosis were inconsistent among different races. Compared with White people, Black people had lower FIB-4 and higher NFS, with *P* < 0.001. Comparing the five indexes in the population with different years of education years, the result showed that there was more significant difference in the relevant indexes of Hepatic steatosis level, indicating that people with years of education ≥15 years have lower hepatic steatosis score, *P* < 0.05. PIR only shows its role in FIB-4 score. The results showed that compared with poor people, FIB-4 index of relatively rich people also increased, *P* < 0.001, which may be related to abnormal hepatic metabolism due to excess nutrition. In the comparison of groups with different smoking conditions, it was found that the group who quit smoking had the highest NAFLD-related index, while the group who did not smoke had the lowest level of most indicators (*P* < 0.05). However, the five indexes of light drinkers were significantly lower than those of those who did not drink at all, *P* < 0.05.

### Partial correlation analysis of heavy metals, SII and NAFLD correlation index

**Figure 2:**
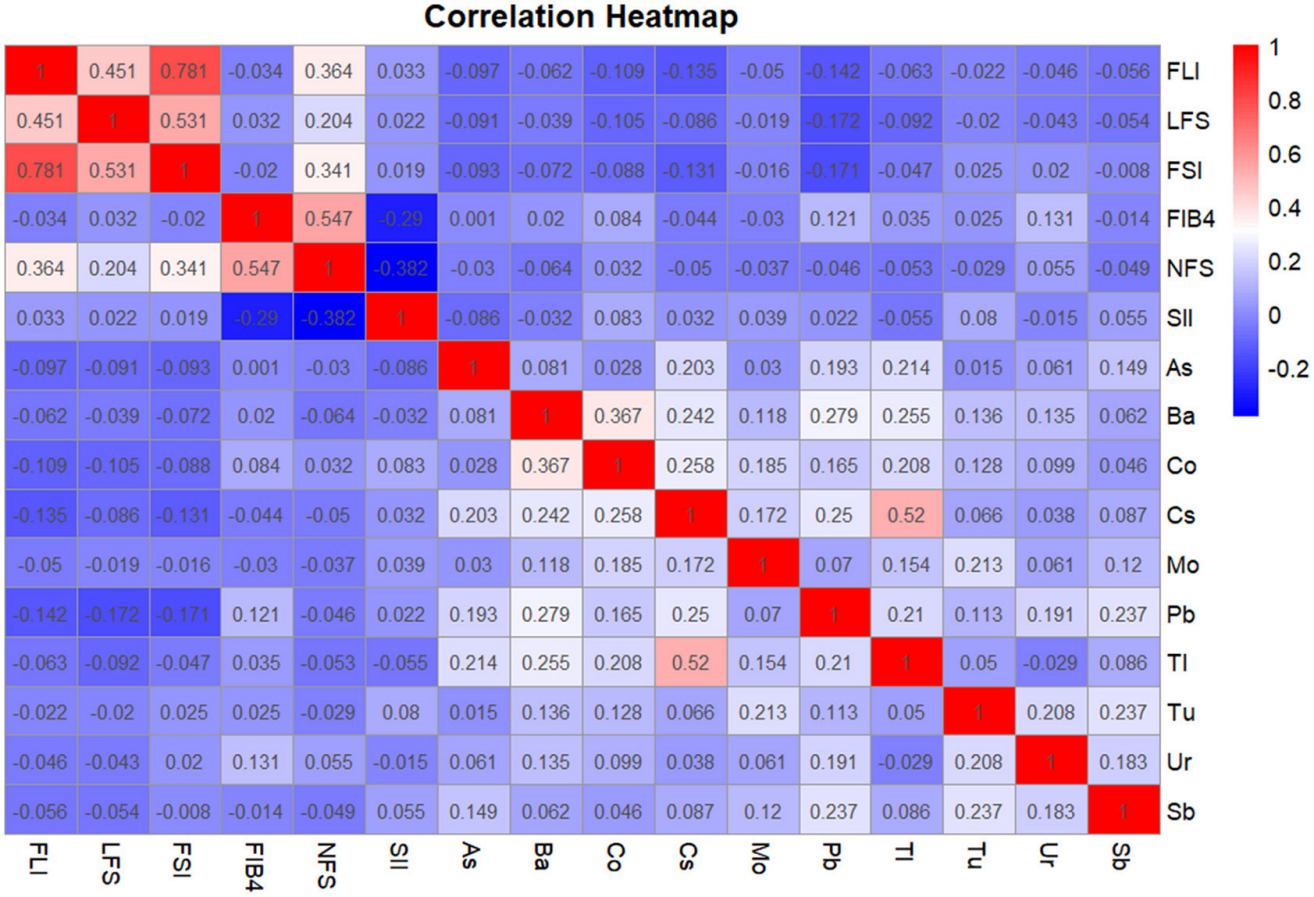
Partial correlation analysis of heavy metals, SII, and NAFLD correlation index. The covariates included sex, age after grouping, race, marriage, education years, smoking, Alcohol drinking, and PIR. The correlation coefficients are shown in color. **Blue** is negatively correlated **red** is a positive correlation. The darker the color, the stronger the correlation. When conducting the overall analysis of heavy metals, logarithmic transformed values are all adopted.

In order to accurately compare the correlation between heavy metals, SII and NAFLD correlation index, we used partial correlation analysis to calculate this, and 8 covariables including age, gender, race, education years, marry, PIR, smoke and drink were controlled. The results were shown as a heat map in Figure 2. Generally speaking, most of the 10 heavy metals were positively correlated with each other. SII appeared to be more closely associated with hepatic fibrosis, with FBI-4 (*r* = −0.290, *P* < 0.001) and NFS (*r* = −0.382, *P* < 0.001), but not with steatosis. There was no positive correlation between heavy metals and hepatic steatosis index FLI, LFS, and FSI, indicating that Co, Pb, Ur were positively correlated with FIB-4, and the correlation coefficients were *r* = 0.084(*P* = 0.036), *r* = 0.121(*P* = 0.002), *r* = 0.131(*P* = 0.001), respectively. Among them, this might be related to heavy metal poisoning after which the induced oxidative stress response may preferentially trigger hepatic fibrosis, It was not associated with steatosis (44). Comparing the correlation between heavy metals and SII, the results showed that Co (*r* = 0.083, *P* = 0.037) and Tu (*r* = 0.080, *P* = 0.044) were significantly positively correlated with SII.

### Associations of FIB-4, NFS, and SII with heavy metals by GLM models

**Figure 3:**
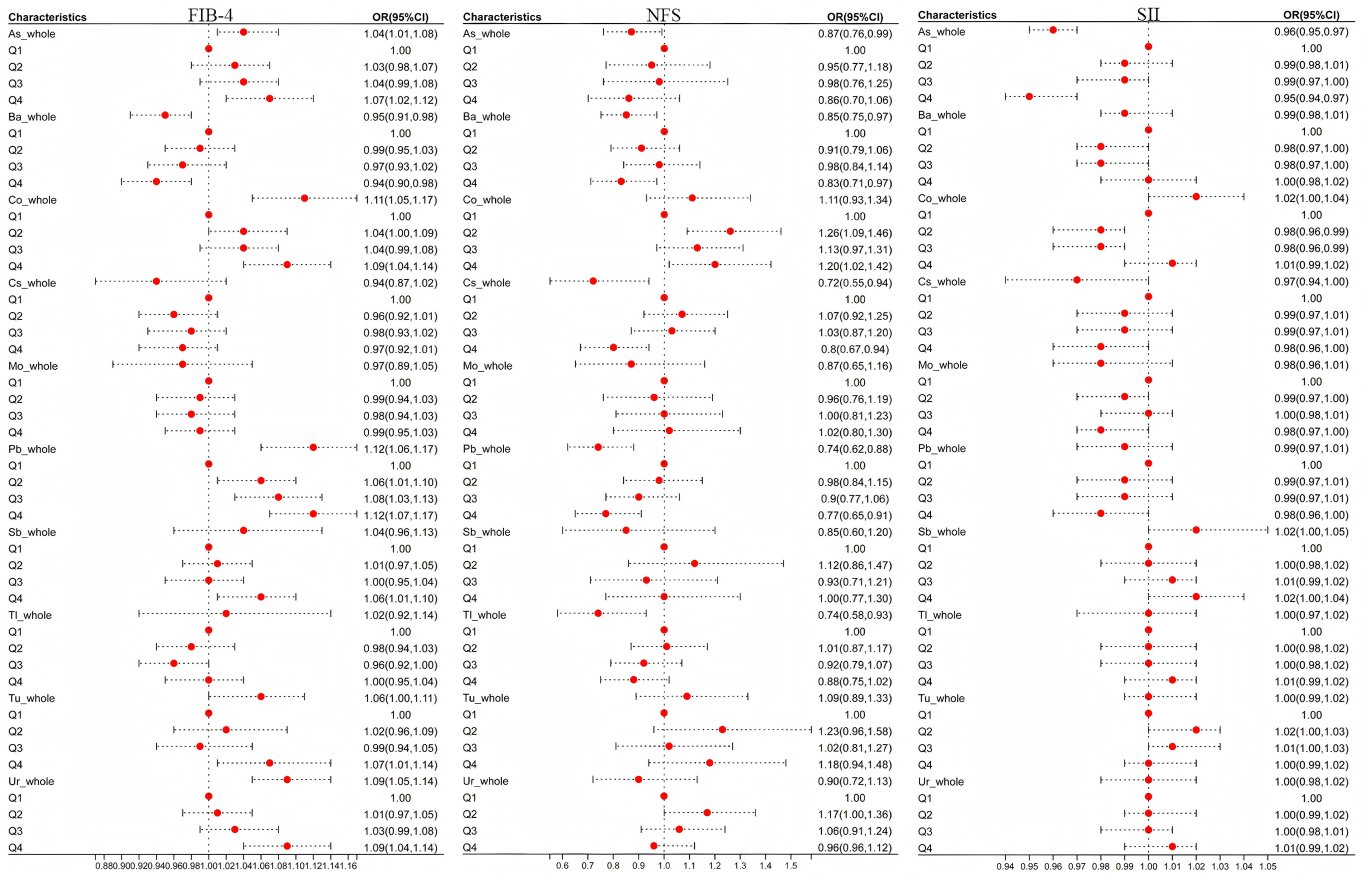
The influence of heavy metal exposure on FIB-4,NFS, and SII. OR was the odds ratio calculated by weighted linear regression after adjusting for age, sex, race, education years, marry, PIR, smoke, and drink. When conducting the overall analysis of heavy metals, logarithmic transformed values are all adopted, When comparing the correlation between the overall level and hepatic fibrotic markers, *P* < 0.05 was taken as the criterion; Taking Q1 as the reference, when comparing the associations of Q2, Q3, and Q4 with hepatic fibrous markers, correction was made according to Bonferroni, with *P* < 0.0125(0.05/4) as the standard.

Since SII showed no significant correlation with hepatic steatosis indices (FLI, LFS, FSI), further analysis was performed only on hepatic fibrosis indices (FIB-4, NFS). The results were illustrated in Figure 3. Analyzing each heavy metal's overall association with SII revealed that As (OR = 0.96, 95% CI = [0.95–0.97], *P* < 0.001) and Cs (OR = 0.97, 95% CI = [0.94, 1.00], *P* = 0.039) showed significant negative correlations, while Sb (OR = 1.02, 95% CI = [1.00, 1.05], *P* = 0.016) exhibited a positive correlation. When taking Q1 as the reference and comparing the correlations between Q2, Q3, Q4 and SII, As (Q4: OR = 0.95, 95% CI = [0.94–0.97], *P* < 0.001) and Co (Q2: OR = 0.98, 95% CI = [0.96–0.99], *P* = 0.011; Q3: OR = 0.98, 95% CI = [0.96–0.99], *P* = 0.009). The negative correlation with SII was highlighted (*P* < 0.0125). This may be related to the fact that arsenic-induced oxidative stress can damage immune cells and promote the proliferation of T lymphocytes, thereby reducing SII (46).

Subsequent focus on As, Co, Cs, and Sb in FIB-4/NFS analyses demonstrated that at overall levels, As (OR = 1.04, 95% CI = [1.01, 1.08], *P* = 0.012) and Co (OR = 1.11, 95% CI = [1.05, 1.17], *P* < 0.001) were positively associated with FIB-4. Quartile regression maintained these positive associations (*P*_As_Q4_ = 0.002, *P*_Co_Q4_ <0.001). Conversely, in NFS analyses, As and Cs showed negative correlations at overall levels, while quartile analysis revealed Co's positive association (*P*_Co_Q2_ = 0.002 <0.0125) and Cs's persistent negative correlation (*P*_Cs_Q4_ = 0.007 <0.0125) with NFS.

### WQS regression model to assess the association of heavy metal co-exposure with FIB-4, NFS, and SII

After exposure to heavy metals, it often showed the combined effect of multiple metals rather than a single metal acting alone. After adjusting for covariates, the combined effect of heavy metals was significantly positively correlated with FIB-4 and SII (*P*_FIB − 4_ <0.001, *P*_SII_ <0.001), but not correlated with NFS (Table 2).

**Table 2:** The association between WQS index of combined heavy metal exposure and FIB-4, NFS, and SII.

**Indicator list**	**Categorical**	**Estimate**	**Std. error**	***t***	***P***
FIB-4	Model 1	0.209	0.014	14.600	<0.001
	Model 2	0.067	0.018	3.800	<0.001
NFS	Model 1	0.079	0.056	1.420	0.157
	Model 2	−0.069	0.049	−1.420	0.156
SII	Model 1	0.029	0.006	4.900	<0.001
	Model 2	0.023	0.006	3.600	<0.001

Model 1 was the crude regression before adjusting for covariates; Model 2 was the regression model for adjustment age, gender, race, education years, marry, PIR, smoke, and drink.

**Figure 4:**
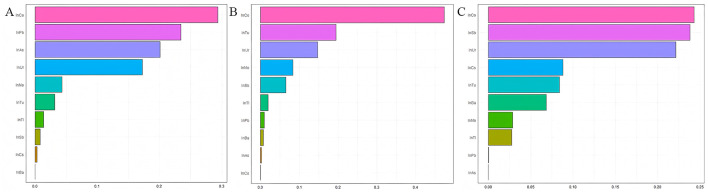
Estimated weights assigned to each exposure based on WQS regression modeled in the positive direction with respect to the FIB-4, NFS, and SII. **(A)** Estimated weights assigned for FIB-4; **(B)** Estimated weights assigned for FIB-4; **(C)** Estimated weights assigned for FIB-4; Models were adjusted for age, gender, race, education years, marry, PIR, smoke and drink.

In Figure 4, the weights of each metal in the combined exposure were presented. The results showed that Co has the greatest impact on FIB-4, NFS, and SII.

### Restricted Cubic Spline analysis was conducted to explore the relationship between As, Co, Ur, and FIB-4, NFS

**Figure 5:**
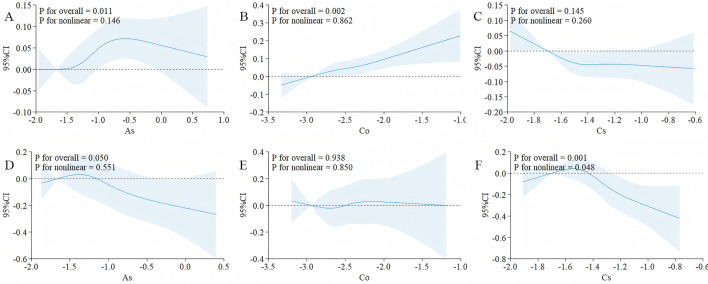
After adjusting for covariates, the correlations between As, Co and Cs with FIB-4 and NFS were evaluated using RCS. **(A)** The correlations between As and FIB-4; **(B)** the correlations between Co and FIB-4; **(C)** the correlations between Cs and FIB-4; **(D)** the correlations between As and NFS; **(E)** the correlations between Co and NFS; **(F)** the correlations between Cs and NFS; The covariates included sex, age after grouping, race, marriage, education years, smoking, Alcohol drinking; The solid black lines correspond to the central estimates, and the gray-shaded regions indicate the 95% confidence intervals; When conducting the overall analysis of heavy metals, logarithmic transformed values are all adopted.

Based on the results of weighted linear regression and WQS, the associations of As, Co, and Cs with FIB-4 and NFS were screened out. Further RCS analysis was conducted, and the results was shown in the Figure 5. The overall analysis showed that there was a statistically significant association between As and FIB-4, *P* = 0.011, and the non-linear relationship holds (*P* = 0.146 > 0.05), indicating that high-concentration exposure to As was a risk factor for hepatic fibrosis.

A significant association was observed between cobalt (Co) and the FIB-4 index (*P*_overall_ = 0.002), with the relationship best described by a linear model (*P*_non − linear_ = 0.862). Similarly, cesium (Cs) showed a significant association with the NFS score (*P*_overall_ = 0.001), and the relationship also followed a linear trend (*P*_non − linear_ = 0.048).

### The mediation analysis of SII on the relationship between As, Co Cs, and FIB-4, NFS

**Figure 6:**
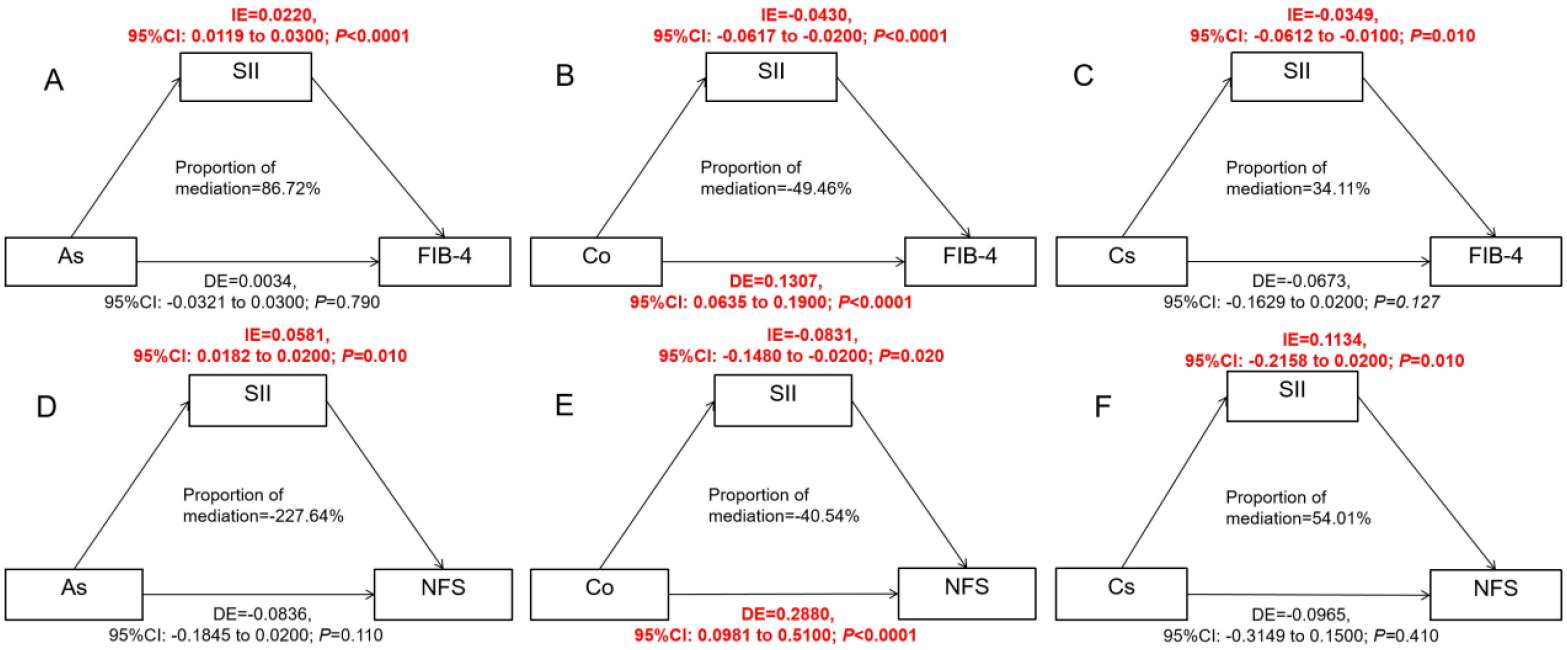
Estimate the association ratio among SII-mediated As, Co, Cs, and FIB-4, NFS. **(A–C)** were for analyzing the effects of As, Co and Cs on FIB-4 respectively; **(D–F)** were to analyze the influences of As, Co and Cs on NFS respectively. The model has been adjusted according to age, gender, race, education years, marry, PIR, smoke and drink; SII, As, Cs, and Co have all undergone logarithmic transformation; The mediation effect is calculated using the “Mediation” package. When conducting the overall analysis of heavy metals, logarithmic transformed values are all adopted.

The result of the mediating effect is presented in Figure 6. After adjusting for confounding factors, SII showed a mediating role in the associations among As, Co, Cs, and FIB-4, and the β-values of its mediating effect were 0.0220 (95% CI: 0.0119, 0.0300) and −0.0430 (95% CI:), respectively. −0.0617, −0.0200) and −0.0349 (95% CI: −0.0612, −0.0100) (Figures 6A–C). The mediating effect ratios were 86.73% and 34.11%, respectively, indicating that SII successively explained 86.73% and 34.11% of the effects of As and Cs on fibrosis. Meanwhile, SII inhibited the positive effect of Co on FIB-4 (49.46%) and NFS (40.54%). If SII was ignored, the effect of Co would be underestimated.

After covariate adjustment, SII mediated the associations between As, Co, Cs and NFS, and the β values of its mediating effect were 0.0581 (95% CI: 0.0182, 0.0200) and −0.0831 (95% CI: −0.1480, −0.0200) respectively and 0.1134 (95% CI: −0.2158, 0.0200) (Figures 6D–F).

## Discussion

To our knowledge, this is the first study to evaluate the mediating role of SII in heavy metal-induced non-alcoholic hepatic injury. With global industrialization, toxic pollutants (e.g., heavy metals) are increasingly released into the environment, accumulating in air, soil, drinking water, and food, ultimately leading to human exposure and tissue damage (47, 48). Existing epidemiological and experimental studies support the role of toxic metal exposure in NAFLD (49), primarily through mechanisms such as oxidative stress, endoplasmic reticulum (ER) stress, pyroptosis, ferroptosis, and dysregulated autophagy (50).

In this study involving 5,613 participants, SII was mainly significantly negative associated with the indicators FIB-4 and NFS representing hepatic fibrosis, rather than hepatocellular steatosis, and although As (arsenic) and Co reduced SII, they ultimately promoted the formation of hepatic fibrosis. Cs was shown to inhibit the occurrence of hepatic fibrosis by reducing SII.

Regarding the association between SII and hepatic fibrosis, the current research results are not completely consistent. This might be caused by the differences in the selected research population. Ma et al. found that a higher SII had a higher positive correlation with the risk of FIB-4 [OR (95% CI): 5.69 (2.17–14.90), *p* < 0.001] (51). However, an analysis of adults with NHANES published in 2022 did not find an association between SII and hepatic fibrosis (52). The results published in 2024 showed that SII was significantly negatively correlated with hepatic fibrosis in the entire population and the diabetic population (53, 54), and the conclusion of this study is consistent with this. The inconsistent research results may, on the one hand, stem from the included population. In this study, the focus was on the population exposed to heavy metals. Heavy metal exposure causes apoptosis of immune cells through pathways such as oxidative stress (55) and DeoxyriboNucleic Acid (DNA) methylation (56), thereby reducing the level of SII. On the other hand, with the increase of heavy metal exposure, the disease progresses to the stage of hepatic fibrosis, and the blood content in the body significantly decreases, mainly lymphocytes, thereby significantly reducing the SII level and presenting a negative correlation phenomenon (53).

In this study, As (arsenic) was negatively correlated with SII, that is, higher As levels were associated with lower SII values. The weighted group regression analysis for FIB-4 indicated that low-dose As did not induce hepatic fibrosis. However, when the body was exposed to high-dose As, hepatic fibrosis became obvious, and SII played a major positive mediating role in this process. This indicates that low doses of arsenic are harmless to physical health, while high concentrations of arsenic exposure cause hepatic damage. Based on the conclusion that low doses was harmless, arsenic preparations (As) could be used in disease treatment, such as the treatment of acute myeloid leukemia (57). This phenomenon could be attributed to the reduced expression of arsenic transporter aquaporin-9 (AQP9), which led to a decrease in intracellular arsenic accumulation and reduced the sensitivity of these cells to arsenic trioxide (ATO) treatment (58). These findings were consistent with the anti-tumor and immunomodulatory effects of ATO in autoimmune diseases, and excessive use of ATO could lead to hepatotoxicity, nephrotoxicity and cardiotoxicity (59).

In 2017, Gao et al. discovered that ATO reduces the percentage of myeloid-derived suppressor cells (MDSCs) in the spleen, weakening their immunosuppressive effect on T cells and thereby promoting T-cell proliferation and immunoregulation (60). Additionally, As suppresses bone marrow hematopoiesis by reducing GATA-2 DNA-binding activity, inhibiting the proliferation and differentiation of neutrophil precursor cells (61). In summary, arsenic exposure promotes neutrophil apoptosis while enhancing lymphocyte proliferation, ultimately leading to the negative correlation between As and SII. When As accumulates beyond a certain threshold, it decreases the activity of antioxidant enzymes, significantly increasing oxidative stress, inflammation, DNA damage, and apoptosis, contributing to the development of various diseases, including hepatic injury (62, 63).

Cobalt(Co) was also a relatively important heavy metal in this study, due to its dual characteristics. It is not only an indispensable trace element in the human body, but also can cause damage to the body when excessive. Co has become increasingly prevalent in daily life with the development of new electric vehicles, as a cathode material for batteries (64). In this study, Co was identified through WQS (weighted quantile sum) analysis. Correlation analysis revealed significant positive associations between Co and both FIB-4 and SII. Further mediation analysis demonstrated that SII exerted a partial negative mediating effect in the relationship between Co and FIB-4/NFS. Co can induce inflammatory responses *in vivo* through oxidative stress. Additionally, it promotes a HIF-1α-dependent metabolic shift from oxidative phosphorylation to glycolysis in macrophages, which plays a crucial role in activating inflammatory responses (65), thereby disrupting normal immune function (66). High concentrations of Co may lead to excessive inflammatory responses, endocrine disruption, adverse developmental effects, and even mortality (67), findings that are consistent with the results of this study (68).

Initially, cesium (Cs) attracted attention due to its radioactive properties (69). Recent studies had demonstrated that its isotope, cesium-131 (^131^Cs), could be encapsulated in seeds or microspheres and implanted into tumors (e.g., prostate cancer), delivering localized high-dose radiation while minimizing damage to surrounding healthy tissues, thus enabling its application in treating brain, prostate, and head and neck cancers (70–72). However, research on the association between Cs and hepatic fibrosis remains limited. In 2020, Ziasmin Khatun et al. co-cultured NIH/3T3 mouse fibroblasts with metal chlorides (Li, Na, K, Rb, and Cs) and found that Cs suppressed fibroblast proliferation and migration (73). The cellular effects of Cs might be related to intracellular metabolism. Given its similar ionic radius to potassium (K^+^), Cs could permeate potassium channels—sometimes even with higher selectivity than K^+^ itself (74), which potentially disrupting normal cellular metabolism by interfering with potassium uptake (75, 76). In our study, Cs exhibited a negative correlation with both the systemic immune-inflammation index (SII) and hepatic fibrosis. Notably, SII demonstrated a positive mediating effect in their association, which aligns with the aforementioned findings. These results suggested that Cs may influence fibrotic processes through immunometabolic pathways, warranting further investigation into its mechanistic role in hepatic fibrosis.

This study possesses several notable strengths. First, the data were derived from the National Health and Nutrition Examination Survey (NHANES) conducted by the Centers for Disease Control and Prevention (CDC), representing the highest quality of experimental results. Second, to our knowledge, this is the first study to comprehensively analyze the associations between heavy metal exposure and multiple NAFLD-related indicators of hepatic steatosis (including FLI, LFS, and FSI) as well as hepatic fibrosis markers (FIB-4 and NFS). Furthermore, we employed mediation analysis to evaluate the potential mediating role of SII in these relationships. However, several limitations should be acknowledged. The cross-sectional design of our study precludes the establishment of causal relationships between the examined variables. Future longitudinal studies are warranted to validate these observations and elucidate the underlying mechanisms.

## Conclusions

In this study, there was a significant correlation of As and Co between heavy metals and hepatic fibrosis indicators FIB-4 and NFS, and SII played a mediating role in this association. Cs was significantly negatively correlated with SII and hepatic fibrosis indicators, and SII played a positive mediating role in this association.

## Data Availability

The original contributions presented in the study are included in the article/supplementary material, further inquiries can be directed to the corresponding author.
